# Optimizing *Pneumocystis jirovecii* Prophylaxis in Immunocompromised Patients: Defining the Clinical Role of Atovaquone

**DOI:** 10.1111/tid.70255

**Published:** 2026-06-02

**Authors:** To N. Pham, Kelly A. Cairns, Adithi Ramachandra, Susan G. Poole, Carmela E. Corallo, Bradley J. Gardiner

**Affiliations:** ^1^ Department of Pharmacy Alfred Health Melbourne Victoria Australia; ^2^ Department of Infectious Disease Alfred Health Melbourne Victoria Australia; ^3^ School of Translational Medicine Monash University Melbourne Victoria Australia

**Keywords:** atovaquone, immunocompromised, *Pneumocystis jirovecii*, prophylaxis, transplant

## Abstract

**Background:**

*Pneumocystis jirovecii* pneumonia (PJP) is associated with poor outcomes in immunocompromised patients. Cotrimoxazole is the first‐line prophylactic agent, with atovaquone an alternative. At our center, atovaquone use considerably increased after 2020. The aims of this retrospective cohort study were to understand the usage of atovaquone over time and explore the reasons for this increase.

**Methods:**

Patients receiving atovaquone for PJP prophylaxis from 2015 to 2022 were included. Clinical data were extracted to characterize atovaquone prescribing patterns and cotrimoxazole contraindications, with comparisons made between patients before 2020 and those afterward.

**Results:**

Here, 149 patients were included, 24 (16%) before 2020 and 125 (84%) after 2020. The cohort comprised solid organ transplant recipients (49%), patients with hematologic conditions (35%), and human immunodeficiency virus (5%). Reasons for not prescribing cotrimoxazole included cytopenias (37%), renal impairment (15%), and drug interactions (12%). Median duration was significantly longer after 2020 (6.0 vs. 2.3 months, *p* = 0.047). Before 2020, most patients (71%) had underlying hematologic disease, but after 2020, solid organ transplant recipients accounted for 60%. Renal impairment appeared as a contraindication to cotrimoxazole only post‐2020. After atovaquone, 67 patients transitioned to an alternative, most commonly cotrimoxazole (56/67, 84%). There were no breakthrough PJP cases.

**Discussion:**

Atovaquone is an alternative PJP prophylaxis agent, used predominantly when cotrimoxazole is not tolerated. Increased usage was related to broader indications, uptake in new patient groups, difficulty tolerating or accessing alternatives, and longer durations. While atovaquone appears safe and effective, regular review of patients receiving it could facilitate reassessment of the initial indication, aiming to reinstate cotrimoxazole whenever appropriate.

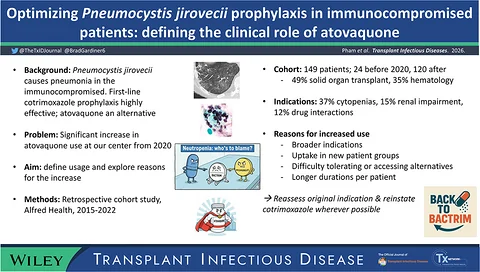

## Graphical abstract

In a retrospective cohort study of 149 patients receiving atovaquone for PJP prophylaxis, increased usage was related to broader indications, uptake in new patient groups, difficulty tolerating or accessing alternatives, and longer durations used per patient. Regular reassessments can return many patients successfully to cotrimoxazole, the first‐line choice wherever possible.

## 1 Background

*Pneumocystis jirovecii* is a fungal pathogen known to cause life‐threatening pneumonia in immunocompromised patients, particularly those with solid organ transplants, human immunodeficiency virus (HIV), and hematologic malignancies. Before the implementation of routine *Pneumocystis jirovecii* pneumonia (PJP) prophylaxis, the risk among immunocompromised patients without HIV was approximately 5%–15% [1]. Consequences can be severe, including hypoxic respiratory failure with reported mortality ranging from 33% to 69% [2].

Antimicrobial prophylaxis is highly effective at preventing PJP [3]. International guidelines recommend the use of cotrimoxazole (trimethoprim‐sulfamethoxazole) as first line [4, 5]. Cotrimoxazole is the most effective agent against PJP [6] and additionally provides coverage for toxoplasmosis [7, 8, 9] and *Nocardia* spp [10]. Cotrimoxazole is generally well‐tolerated; however, several adverse effects have been reported, including leukopenia, hyperkalemia, renal impairment, and gastrointestinal disturbances [11, 12, 13, 14, 15]. Sulfonamide allergies, which range in severity from mild to life‐threatening, also limit use. Alternative agents, including dapsone and inhaled pentamidine, are available when cotrimoxazole cannot be used; however, each comes with its own risk of adverse effects. Atovaquone, a ubiquinone analogue, has efficacy against PJP; however, it is usually considered a third‐ or fourth‐line option. Atovaquone is less effective than cotrimoxazole for PJP prophylaxis in patients with HIV, but has similar efficacy to inhaled pentamidine or dapsone [16]. The oral suspension formulation of atovaquone makes it easier to administer compared to inhaled pentamidine and has a more favorable side effect profile than dapsone; however, high cost and a lack of accessibility limit its use.

PJP prophylaxis protocols at our institution vary between clinical units, but dapsone and inhaled pentamidine are generally recommended as alternatives to cotrimoxazole, with atovaquone being reserved as the last option. Despite this, from 2020 onward, there was a substantial increase in the use of atovaquone at our center. Therefore, this project aimed to describe the usage of atovaquone over time, understand prescribing patterns and indications, and explore reasons for increased use since 2020.

## 2 Methods

### 2.1 Setting

This study was performed at Alfred Health in Melbourne, Victoria, Australia, a large tertiary referral hospital with approximately 600 acute inpatient and 62 intensive care beds. Our center offers a wide range of services, including heart, lung, kidney, and bone marrow transplants; HIV management; and treatment of other immunosuppressive conditions. Each specialty has antimicrobial prophylaxis protocols tailored to its individual population. There is an established active infectious diseases service, which provides both direct clinical consultation and input into protocol development. The study was reviewed and approved by the Human Research and Ethics Committee at Alfred Health (project number 109/24), with informed consent not required due to its retrospective nature and minimal risk.

### 2.2 Study Design and Population

A retrospective cohort study was undertaken in adult patients managed at our center who commenced atovaquone from January 2015 to December 2022. Patients were included if atovaquone was dispensed through our hospital inpatient or outpatient pharmacy at least once and excluded if they were under 18 years old at the time of atovaquone initiation, received atovaquone for treatment rather than prophylaxis, or if there were insufficient data in the medical records.

### 2.3 Data Collection and Definitions

Clinical data were collected from electronic medical records. This included basic demographics, atovaquone indication, dose and duration, prescribing team, reasons for not using cotrimoxazole, and preceding and subsequent PJP prophylaxis agents. Patients were censored at death, the day of atovaquone discontinuation, loss to follow‐up, or the end of the study period if atovaquone was still ongoing. The atovaquone initiation date was defined as the date atovaquone was first dispensed from our pharmacy, and the discontinuation date was defined as either the first documented completion date in the medical records or the point at which the dispensed supply would reasonably have been exhausted.

Atovaquone dispensing data were obtained from the hospital dispensing software (iPharmacy version 12.0.0, Dedalus Australia). For comparison, dispensing data were also obtained for pentamidine, but were unavailable for cotrimoxazole or dapsone, as many patients obtained this outside our center. Antimicrobial utilization was measured using World Health Organization‐defined daily doses (DDDs), which were 2.25 g for atovaquone and 0.28 g for pentamidine.

### 2.4 Statistical Analysis

Patients from 2015 to 2019 were compared to patients from 2020 to 2022. Categorical data were reported as counts and percentages, continuous data as means ± standard deviations if normally distributed and medians with interquartile range if non‐normal. Groups were compared using the chi‐squared/Fisher's exact test for categorical variables, Student's *t*‐test for normally distributed variables, and the Wilcoxon rank sum test for non‐normally distributed continuous variables. All analyses were performed with the R statistical software platform version 4.4.2 (RStudio version 2024.12.0+467).

## 3 Results

### 3.1 Cohort Description

Between 2015 and 2022, a total of 156 patients received at least one bottle of atovaquone through the hospital pharmacy. Seven patients met our exclusion criteria, leaving 149 patients in our final cohort (Figure S1). Just over half of the included patients were male (81/149, 54%) with a mean age at atovaquone commencement of 58 ± 13 years (Table 1). Atovaquone was most frequently prescribed in lung transplant recipients (35/149, 23%), followed by kidney transplant (22/149, 15%), heart transplant (14/149, 9%), bone marrow transplant (30/149, 20%), or other hematologic conditions (24/149, 16%). The median duration of atovaquone use was 4.6 months (IQR: 1.6–14.7 months).

**TABLE 1:** Characteristics of the 149 patients initiating atovaquone in our cohort.

Characteristic	Details (*n* = 149)
Male gender, no. (%)	81 (54%)
Age (years), mean ± SD	58 ± 13
Main comorbidity, no. (%) Bone marrow transplant	30 (20%)
Other hematology	24 (16%)
HIV	8 (5%)
Heart transplant	14 (9%)
Kidney transplant	22 (15%)
Lung transplant	35 (23%)
Multi‐organ transplant	5 (3%)
Other	11 (7%)
Atovaquone duration (months), median, IQR	4.6, 1.6–14.7
Agent before atovaquone, no. (%) Cotrimoxazole	66 (44%)
Pentamidine	27 (18%)
Dapsone	32 (21%)
None	20 (13%)
Trimethoprim	4 (3%)
Reasons for not using cotrimoxazole, no. (%) Cytopenias	54 (36%)
Renal impairment	22 (15%)
Drug interaction	18 (12%)
Rash	17 (11%)
Multiple reasons	15 (10%)
Severe allergy	7 (5%)
Other	16 (11%)
Reason for changing pentamidine to atovaquone (*n* = 27), no. (%) Administration difficulties	12 (44%)
Flu‐like symptoms	7 (26%)
Unknown	4 (15%)
Other	4 (15%)
Reasons for changing dapsone to atovaquone (*n* = 32), no. (%) Hemolysis	11 (34%)
Methemoglobinemia	10 (31%)
Rash	3 (9%)
Other	8 (25%)
Outcomes after atovaquone, no. (%) Changed to an alternative drug	55 (37%)
Intolerance, changed to alternative	12 (8%)
Intolerance, ceased prophylaxis	14 (9%)
Ceased prophylaxis, no ongoing PJP risk	24 (16%)
Prescriptions not refilled	13 (9%)
Death	20 (13%)
Ongoing at censoring	11 (7%)
Agent following atovaquone (*n* = 67), no. (%) Cotrimoxazole	56 (84%)
Pentamidine	8 (12%)
Dapsone	2 (3%)
Trimethoprim	1 (1%)

The most common reasons for prescribing atovaquone instead of cotrimoxazole were cytopenias (36%), renal impairment (15%), and drug interactions (12%). Most patients transitioned to atovaquone from cotrimoxazole (66/149, 44%), with smaller numbers transitioned from dapsone (21%) and pentamidine (18%). Atovaquone was used as the initial PJP prophylaxis agent for 20 patients (13%). Among those switched from dapsone, the predominant reasons were hemolysis (11/32, 34%) and methemoglobinemia (10/32, 31%), while administration difficulties accounted for most transitions from pentamidine (12/32, 44%).

After the atovaquone course, 24 patients (16%) ceased prophylaxis completely as they were no longer considered at risk for PJP. A total of 67 patients (46%) were changed to a different agent, with cotrimoxazole being the most common choice (56/67, 84%). Atovaquone was ceased due to adverse effects, such as gastrointestinal discomfort and taste disturbances in 26 patients (17%), and prescriptions were not refilled in 13 patients (9%) despite prescribers’ intention to continue it. There were no reported cases of breakthrough PJP infection while patients were receiving atovaquone.

### 3.2 Prescribing Trends Before and After 2020

Of the 149 patients included, 24 (16%) commenced atovaquone between 2015 and 2019, and 125 (84%) between 2020 and 2022 (Table 2). The two time periods differ notably in underlying comorbidities, atovaquone duration, and reasons for avoiding cotrimoxazole. Before 2020, the majority of atovaquone patients had underlying hematologic disease (17/24, 71%). In contrast, from 2020 to 2022, solid organ transplant recipients accounted for 60% (75/125) of patients (*p* < 0.001, Figure S2). Renal impairment, which was not recorded as a reason for avoiding cotrimoxazole in the earlier period, was documented in 22 patients between 2020 and 2022 (Figure S3). The duration of atovaquone prophylaxis also increased significantly from a median of 2.3 months before 2020 to a median of 6.0 months after 2020 (*p* = 0.047, Figure S4). Following completion of atovaquone, 50% of patients in the 2015–2019 cohort were switched to cotrimoxazole compared with 91% in the 2020–2022 cohort (*p* < 0.05).

**TABLE 2:** Characteristics of patients initiating atovaquone before 2020 compared to those starting after 2020 (*n* = 149).

Characteristic	2015–2019(*n* = 24)	2020–2022(*n* = 125)	*p* value
Male gender, no. (%)	11 (46%)	70 (56%)	0.4
Age (years), mean ± SD	52 ± 15	59 ± 13	0.048
Main comorbidity, no. (%) Hematologic (including BMT)	17 (71%)	37 (30%)	< 0.001
Solid organ transplant	1 (4%)	75 (60%)	
Other	6 (25%)	13 (10%)	
Atovaquone duration (months), median, IQR	2.3, 0.9–6.1	6.0, 1.7–15.9	0.047
Agent before atovaquone, no. (%) Cotrimoxazole	5 (21%)	61 (49%)	0.064
Pentamidine	7 (29%)	20 (16%)	
Dapsone	8 (33%)	24 (19%)	
None	4 (17%)	16 (13%)	
Trimethoprim	0	4 (3%)	
Reasons for not using cotrimoxazole, no. (%) Cytopenias	8 (33%)	46 (37%)	0.055
Renal impairment	0	22 (18%)	
Drug interaction	2 (8%)	16 (13%)	
Allergy Other	6 (25%) 8 (33%)	18 (14%) 23 (18%)	
Outcomes after atovaquone, no. (%) Ceased completely	8 (33%)	43 (32%)	0.84
Changed to alternative	12 (50%)	55 (44%)	
Death	4 (17%)	16 (13%)	
Ongoing at censoring	0	11 (9%)	
Agent following atovaquone, no. (%) Cotrimoxazole	6 (50%)	50 (91%)	0.002
Pentamidine	5 (42%)	3 (6%)	
Dapsone	1 (8%)	1 (2%)	
Trimethoprim	0	1 (2%)	

### 3.3 Overall Atovaquone and Pentamidine Usage

Atovaquone utilization compared to pentamidine is shown in Figure 1 and Table 3. Annual atovaquone usage remained relatively low from 2015 to 2019, with a median of 504 g/year (IQR: 308–504 g), compared to during 2020–2022, when there was a substantial increase to 4200 g/year (IQR: 2716–5033 g, *p* = 0.04). This corresponded to a ninefold increase in cost from AUS$32 072 per year (IQR: $19 641–$32 666) to AUS$275 154 per year (IQR: $177 889–$329 729, *p* = 0.04). This contrasts with pentamidine outpatient utilization, where annual usage reduced from 196.1 g (IQR: 185.4–210.0 g) in 2015–2019 to 118.9 g (IQR: 88.4–119.5 g, *p* = 0.04) in 2020–2022. Pentamidine inpatient use remained relatively stable.

**FIGURE 1:**
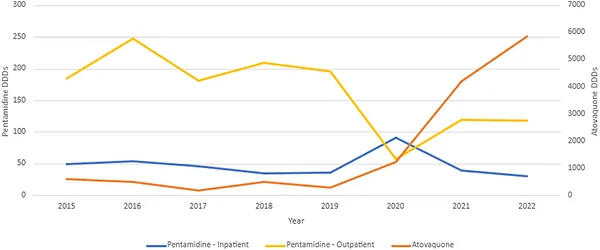
Overall usage of atovaquone and pentamidine from January 2015 to December 2022 in defined daily doses (DDDs).

**TABLE 3:** Annual drug usage in defined daily doses (DDDs) and annual costs in Australian dollars.

Drug	Overall	2015–2019	2020–2022	*p* value
Pentamidine (inpatient), median, IQR	42.9, 36.2–51.4 $17 169, $16 514–$19 363	46.1, 36.4–50.4 $17 060, $14 875–$17 077	39.6, 35.4–65.4 $20 769, 19 014–33 048	1.0 0.07
Pentamidine (outpatient), median, IQR	183.2, 119.7–199.6 $67 839, $59 992–$87 009	196.1, 185.4–210.0 $86 313, $69 714–$89 096	118.9, 88.4–119.5 $63 080, $46 081–$64 522	0.04 0.14
Atovaquone, median, IQR	553, 455–1974 $36 839, $28 964–$129 257	504, 308–504 $32 072, $19 641–$32 666	4200, 2716–5033 $275 154, $177 889–$329 729	0.04 0.04

## 4 Discussion

PJP prevention remains a crucial strategy for improving outcomes in immunosuppressed patients. Cotrimoxazole is the prophylactic agent of choice because of its efficacy against PJP and other opportunistic pathogens, but its use can be limited by intolerance and adverse events. Atovaquone is a relatively safe, effective alternative that overcomes many of the limitations of other prophylactic agents [17]. In this study of 149 patients receiving atovaquone as PJP prophylaxis between 2015 and 2022, we identified a substantial increase in utilization after 2020 due to both an increased number of patients starting as well as a significant increase in atovaquone duration used for each individual patient.

The increase in atovaquone use from 2020 onward coincided with the onset of the COVID‐19 pandemic. Around this time, our hospital implemented restrictions on aerosolized medications in line with international recommendations [18]. It seems likely that difficulties in logistics for pentamidine administration and the favorable side effect profile of atovaquone contributed to the shift in usage. To the best of our knowledge, there are no other published reports of significant increases in atovaquone utilization following the onset of the COVID‐19 pandemic. Despite the early recommendations to avoid aerosolized medications, very few published studies described the pandemic impact on the use of nebulized pentamidine. Liew et al. recognized that restrictions of aerosolized medications during COVID‐19 outbreaks limited their PJP prophylaxis prescribing options and they switched patients from nebulized pentamidine to the intravenous formulation, but did not use atovaquone [19].

The high atovaquone usage at our institution was largely driven by cotrimoxazole adverse events and relative contraindications, including cytopenias and renal impairment. Overall, studies demonstrate that the side effects of cotrimoxazole, specifically neutropenia, occur at very low rates (2%) when used at the standard prophylactic thrice weekly dose [11, 20]. Importantly, some of these adverse effects could be related to underlying comorbidities or concurrent medications, such as valganciclovir and mycophenolate. For example, Zafrani et al. have demonstrated no correlation between cotrimoxazole cessation and duration of neutropenia in a cohort of renal transplant patients [21]. Similarly, in a cohort of 321 heart transplant recipients, rates of hyperkalemia did not decrease when cotrimoxazole was switched to atovaquone [22]. Adverse effects such as cytopenias, hyperkalemia, and elevated creatinine can be transient, and regular review of laboratory results is warranted to determine if cotrimoxazole could be retrialed after atovaquone commencement. Several studies have suggested that use of alternative PJP prophylaxis agents can be minimized through the use of defined thresholds and clear protocols for cessation of cotrimoxazole [14, 23, 24]. McLaughlin et al. demonstrated that subsequent rechallenging of cotrimoxazole post commencement of atovaquone can be successful and is strongly encouraged due to the advantages it provides over other agents [25]. Another critical strategy to improve PJP prophylaxis prescribing is a detailed review of allergies and adverse drug reactions. A small proportion of our patients were not started on cotrimoxazole due to a documented history of rash. There is increasing awareness of the impact and importance of antimicrobial allergy labels as a component of antimicrobial stewardship efforts [26]. Interventions such as the clinical decision tool SULF‐FAST are currently being investigated to develop a user‐friendly pre‐clinic self‐assessment to identify low‐risk allergies, enabling de‐labeling and oral challenge [27]. For patients with certain higher‐risk allergies, cotrimoxazole desensitization protocols are available and have been shown to be a safe, effective, and cost‐saving approach to optimize PJP prophylaxis prescribing [28].

Generally, nebulized pentamidine is less commonly used in practice as it requires specialized equipment and nursing support, and effectiveness depends on its ability to disperse aerosol to all portions of the lungs [29]. Dapsone usage is limited by adverse effects such as oxidative hemolysis and methemoglobinemia, the risk of which can be reduced, but not eliminated, by checking glucose‐6‐phosphate dehydrogenase (G6PD) before starting [23, 30, 31]. Different dosing regimens exist, ranging from 50–100 mg daily to 100–200 mg weekly, and even the weekly dosing has been associated with lower hemoglobin levels than cotrimoxazole in renal transplant recipients [32]. When considering the patient population at risk of PJP, particularly transplant recipients, it is important to note that both dapsone and pentamidine lack activity against toxoplasmosis [33].

Atovaquone is an effective PJP prophylaxis agent, with no breakthrough infections observed in this study, consistent with findings from others [12, 13, 34, 35, 36]. An important limitation is that it lacks activity against *Nocardia* spp., urinary pathogens, and there have been reports of breakthrough toxoplasma infection [37, 38, 39, 40]. In addition, patient adherence and adequate absorption must be considered, particularly as the medication is only available as an oral suspension. In a cohort of adult hematology and HIV‐positive patients, over half of the patients receiving standard dosing (750 mg twice daily) had low plasma concentrations, raising concerns about reduced absorption in immunosuppressed patients with variable and unpredictable bioavailability [41]. Although atovaquone is generally believed to be well‐tolerated, gastrointestinal adverse effects have been reported in up to 10% of patients [42]. Almost one‐fifth of our cohort ceased atovaquone due to adverse effects, including nausea, diarrhea, and taste disturbances. An additional challenge in our setting is that access to atovaquone outside of the hospital pharmacy is limited, and we expect this may reduce adherence. Another advantage of cotrimoxazole is that it is readily available in both tablet and oral suspension, conveniently dosed at three times per week, and the tablets can be easily incorporated into a weekly medication compliance aid (pill/dosette box) if needed.

This study has identified opportunities for improvement in PJP prophylaxis prescribing strategies at our center. First, we noted several instances where atovaquone dispensing records indicated likely prophylaxis interruptions due to irregularities in dispensing. These interruptions were not recognized by treating clinicians, increasing the risk of breakthrough infection. Second, many patients had reported low‐risk cotrimoxazole allergies, presenting an opportunity to integrate cotrimoxazole allergy de‐labeling, oral rechallenge, or desensitization, to enable safe initiation of therapy. Third, guidelines for PJP prophylaxis differed across prescribing units with variable thresholds for ceasing cotrimoxazole. This warrants consolidation of protocols, aiming to standardize criteria for changing cotrimoxazole to alternatives. Fourth, the etiology of laboratory abnormalities attributed to cotrimoxazole, such as cytopenias and renal impairment, should be carefully reviewed, as these can be multifactorial and impacted by comorbidities and concurrent medications such as mycophenolate and valganciclovir. Fifth, regular follow‐up and medication reviews are needed to reassess whether the original indication (e.g., cytopenias) for atovaquone is still present, which would minimize prolonged durations of atovaquone, aiming to reinstate cotrimoxazole for PJP prophylaxis wherever possible. Finally, novel strategies such as using immune biomarkers to individualize risk stratification and newer alternatives to valganciclovir with less marrow toxicity may help in the future [43, 44, 45].

Our study has some limitations that need to be considered when interpreting the results. This was a retrospective, single‐center cohort with statistical power limited by the available sample size. Despite this, as our center comprises a variety of specialized treating units, the patient cohort is largely representative of other major transplant and cancer centers worldwide. Data collection was robust, with individual records reviewed in detail to understand all relevant factors in clinician decision‐making. We relied the clinical documentation that was available, which in some cases was incomplete. Unlike atovaquone and pentamidine, we were unable to reliably obtain dispensing data for cotrimoxazole and dapsone, so we could not analyze overall usage trends of these agents. We also focused solely on the prescribing patterns of atovaquone, so we could not directly compare the effectiveness or safety of atovaquone to other PJP prophylaxis agents. Cost data were available for drug acquisition costs only, so we did not include the costs of nursing support and equipment required for pentamidine administration. Finally, we could not easily identify the larger denominator population at risk for PJP, which may have increased over time.

In summary, PJP is an important opportunistic infection that can affect many immunosuppressed patients and is preventable with antimicrobial prophylaxis. Cotrimoxazole is the first‐line agent of choice with many key advantages. Atovaquone represents a safe and effective alternative; however, its use is limited by high cost, restricted availability, and intolerance, resulting in reduced adherence in some patients. In the meantime, using atovaquone as a short‐term bridge, for example, in the setting of cytopenias, is a good option with regular reviews aiming to reinitiate cotrimoxazole where possible.

## Author Contributions

**Kelly A. Cairns**: conceptualization, writing – review and editing, supervision. **Bradley J. Gardiner**: conceptualization, writing – review and editing, supervision, formal analysis, methodology. **Carmela E. Corallo**: conceptualization, writing – review and editing, supervision. **Adithi Ramachandra**: methodology, validation, formal analysis, software. **To N. Pham**: writing – original draft, data curation. **Susan G. Poole**: writing – review and editing.

## Funding

Bradley J. Gardiner was supported by the Sylvia and Charles Viertel Charitable Foundation Clinical Investigator Award, and a National Health and Medical Research Council (NHMRC) Investigator Grant (2041063).

## Conflicts of Interest

The authors declare no conflicts of interest.

## Supporting information




**Supporting File 1**: tid70255‐sup‐0001‐SuppMat.docx

**Supplementary**: Visual Abstract
