# Supplementary material for: Optimizing Pneumocystis jirovecii Prophylaxis in Immunocompromised Patients: Defining the Clinical Role of Atovaquone
Source: Transpl Infect Dis. 2026 Jun 2;28(5):e70255. doi: 10.1111/tid.70255 (PMC13638778; doi:10.1111/tid.70255)
Supplement: Supplementary file 1 [file TID-28-e70255-s001.docx]

**Optimizing *Pneumocystis jirovecii* prophylaxis in immunocompromised patients: defining the clinical role of atovaquone**

To N. Pham^a^, Kelly A. Cairns^a,b^, Adithi Ramachandra^b^, Susan G. Poole^a^, Carmela E. Corallo^a^, Bradley J. Gardiner^b,c^

^a^Department of Pharmacy, Alfred Health, Melbourne, Victoria, Australia

^b^Department of Infectious Disease, Alfred Health, Melbourne, Victoria, Australia

^c^School of Translational Medicine, Monash University, Melbourne, Victoria, Australia

**Supplementary Figures**

**Figure S1:** Number and flow of study participants.

**Figure S2:** Underlying comorbidities in patients starting atovaquone before and after 2020 (n=149).

**Figure S3:** Reasons why cotrimoxazole was not used in patients commencing atovaquone before and after 2020 (n=149).

**Figure S4:** Duration of atovaquone prophylaxis in patients commencing before (n=24) and after 2020 (n=125).
